# Editorial: Emerging polyoxometalates with biological, biomedical, and health applications

**DOI:** 10.3389/fchem.2022.977317

**Published:** 2022-08-09

**Authors:** Manuel Aureliano, Scott G. Mitchell, Panchao Yin

**Affiliations:** ^1^ Faculdade de Ciências e Tecnologia, Campus de Gambelas, Universidade do Algarve, Faro, Portugal; ^2^ Centro de Ciências do Mar (CCMar), Universidade do Algarve, Faro, Portugal; ^3^ Instituto de Nanociencia y Materiales de Aragón (INMA), Consejo Superior de Investigaciones Científicas-Universidad de Zaragoza, Zaragoza, Spain; ^4^ CIBER de Bioingeniería, Biomateriales y Nanomedicina, Instituto de Salud Carlos III, Madrid, Spain; ^5^ South China University of Technology, Guangzhou, China

**Keywords:** polyoxometalates, antibacterial, antiviral, antidiabetic, biosensors, molecular modulations simulations

The biomedical application of metals, including platinum (Pt), lithium (Li), tungstate (W), gold (Au) or vanadium (V), among others, has become an important and a rapidly growing branch of science (Bertinat et al., 2018; Yeo et al., 2018; Vosahlikova et al., 2020; Ścibior et al., 2020; Silva et al., 2021; Pena et al., 2022; Ochoa, 2022). Besides the well-characterized platinum drugs, bio-active metal-based complexes and clusters, such as gold compounds and polyoxometalates (POMs), as well as metal-based nanoparticles have shown demonstrable anti-cancer, anti-viral and anti-bacterial activities (Bertinat et al., 2018; Yeo et al., 2018; Soria-Carrera et al., 2020; Vosahlikova et al., 2020; Ścibior et al., 2020; Silva et al., 2021; Aureliano et al., 2022a; Pena et al., 2022; Ochoa, 2022; Soria-Carrera et al., 2022). The biological and biomedical application of POMs–in the form of cluster ions, hybrid materials, and POM-based nanoparticles - has tripled in the last decade (Pimpão et al., 2020). In fact, the wide range of POMs uses in medicine may be due to the modulation of several proteins such as aquoporins and P-type ATPases (Gumerova et al., 2018; Fraqueza et al., 2019) although many other biomolecular and biochemical processes are affected by POMs, as illustrated by the well-studied polyoxovanadates (POVs) (Bijelic et al., 2018; Bijelic et al., 2019; Čolović et al., 2020; Aureliano et al., 2021; Aureliano et al., 2022b). POMs against bacteria and in cancer therapy and diagnostics, their modes of action, protein targets and future perspectives were recently reviewed and highlighted (Bijelic et al., 2018; Gumerova et al., 2018; Bijelic et al., 2019; Fraqueza et al., 2019; Čolović et al., 2020; Aureliano et al., 2021; Aureliano et al., 2022b). The majority of the biomedical studies have addressed how POMs affect cancer and bacterial cell growth, not to mention their antiviral activity (Bijelic et al., 2018; Fraqueza et al., 2019; Guedes et al., 2020; Čolović et al., 2020; Aureliano et al., 2021; Aureliano, 2022; Aureliano et al., 2022b), however, much remains to be understood concerning the biochemical mechanism of action of these compounds (Bijelic et al., 2018; Bijelic et al., 2019; Pimpão et al., 2020; Čolović et al., 2020; Aureliano et al., 2021; Aureliano et al., 2022b). The isopolyoxovanadate decavanadate [V10O28]6^−^, {V_10_}, is perhaps the most widely studied POM in biology, showing several roles in key biochemical and cellular processes (Crans et al., 2004; Aureliano, 2009; Aureliano and Crans, 2009; Aureliano et al., 2013; Bijelic et al., 2018; Bijelic et al., 2019; Čolović et al., 2020; Aureliano et al., 2021; Sciortino et al., 2021; Aureliano et al., 2022b). Particularly interesting under the topic of POMs speciation (Gumerova and Rompel, 2020), was the observation that {V_10_} binding to G-actin inhibits its polymerization to F-actin while it prevents {V_10_} decomposition (Ramos et al., 2006). The V_10_/G-actin interaction might interfere with cytoskeleton dynamics and inducing cell morphology changes (Ramos et al., 2006; Sciortino et al., 2021).

**FIGURE 1:**
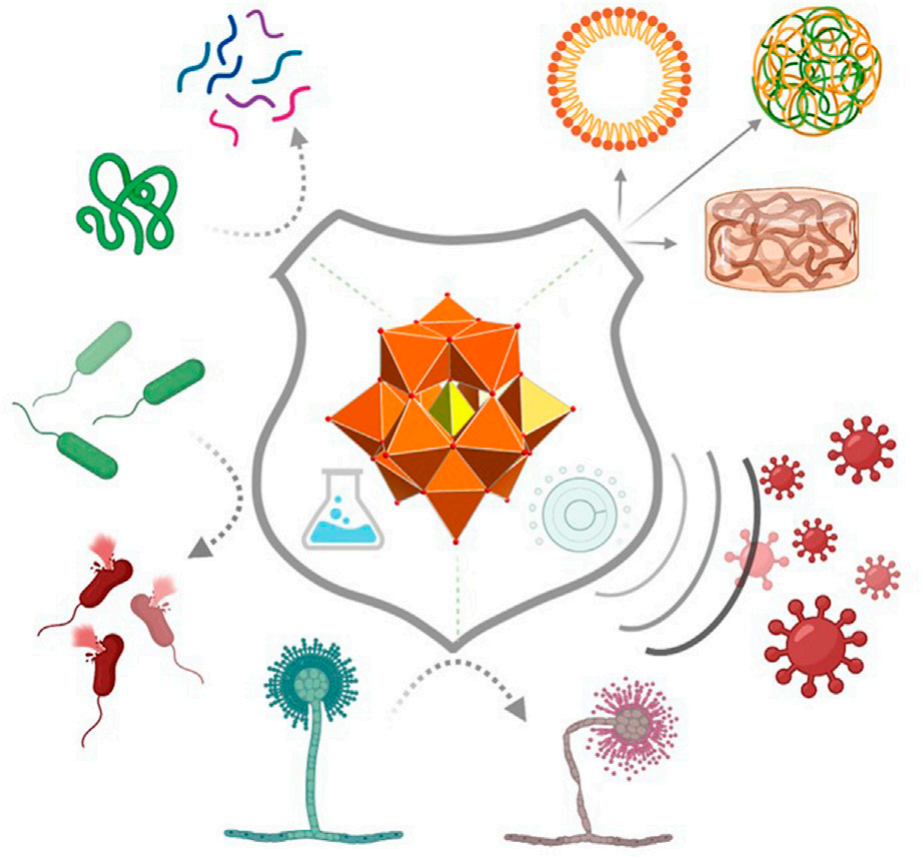
POMs are described with anticancer, antiviral, and antimicrobial activities as well as other biological or biomedical applications such as artificial protease activity (top-left) and self-assembly with biomacromolecules to make micelles, gels & other superstructures (top-right).

Taken in consideration all these effects and properties, it was clear that the future bio-based applications of POMs is bright (Aureliano, 2022). That was why the Guest Editors decided to pursue at Frontiers the promotion and the development of a research topic about “Emerging Polyoxometalates with Biological, Biomedical, and Health Applications” (Figure 1).

Thus, the present Research Topic (RT) aimed to highlight recent advances into *in vitro* and *in vivo* POMs, be they pure, hybrid, or POM-based nanoparticles with anticancer, antiviral, and antimicrobial activities as well as other biological or biomedical applications, such as in diabetes and neurological diseases (Treviño et al., 2019; Treviño and González-Vergara, 2019; Zhao et al., 2019; Atrián-Blasco et al., 2022). Moreover, studies on POMs as biosensors, the redox activity of POMs as sensors for biological factors or markers of specific illnesses were also welcome. Papers addressing biomolecular POMs targets; POMs as ion channels in lipid/cell membranes and transportation of POMs across cell membranes was also welcomed in this Research Topic. The RT had an excellent number of authors (27) that confirmed, immediately after invitation, their participation. However, among others several reasons, directly and/or indirectly COVID has had a major impact on the number of papers submitted, preventing to fulfill, at least in part, some objectives of the present RT.

The first paper published on the present RT Corona-Motolinia et al., by the research group of Professor Enrique González-Vergara from the Benemérita Universidad Autónoma de Puebla (Mexico) in collaboration with coworkers from the Universidad de Granada (Spain). Enrique González-Vergara is a well-known researcher in our scientific community for publishing, for example, several studies about the antidiabetic properties of {V_10_} compounds, particularly metformin-decavanadate (Sánchez-Lombardo et al., 2014; Treviño et al., 2016; Treviño et al., 2019; Treviño and González-Vergara, 2019). The hybrid metformin-decavanadate (Metf-V_10_) (Chatkon et al., 2013) has shown *in vivo* nontoxicological effects on liver and kidney, leading it to be considered as a better treatment for diabetes than metformin (Treviño et al., 2016). However, as suggested previously by Enrique González-Vergara and coworkers, Meft-V_10_ might be also a more effective treatment than metformin in cancer (Sánchez-Lara et al., 2018; Treviño et al., 2019). In fact, herein, the focus of the paper is the antineoplastic activity of another hybrid POVs containing {V_10_}. Among the findings, molecular docking studies with small RNA fragments support the hypothesis that decavanadate’s anticancer activity could be attributed to its interaction with small non-coding RNA molecules.

From the University of Aveiro (Portugal), a review about “Polyoxometalate Functionalized Sensors”, by Veríssimo et al., brings an interesting perspective about POM-based biosensing applications. The University of Aveiro has a tradition in the use of POMs for several different applications (Gamelas et al., 2012; Veríssimo et al., 2017; Veríssimo et al., 2018). In the present review, the authors emphasized that POMs could be used as sensors for detecting and determining molecules and biomolecules in different matrices, many of them with biochemical and clinical relevance, along with analytical figures of merit and main virtues and drawbacks of such devices. Special emphasis is given to the stability of POMs sensitive layers, detection limits, selectivity, and the pH working range.

The third paper of this RT by Long-Sheng Wang from the Hubei University of Technology, Wuhan (China) reports the antiviral activity of POMs. These authors are from a University with previous experience in the use of these derivative POMs against virus (Wei et al., 2020). Herein, it was referred that the covalent linkage between the iodobenzoyldiazenido components and POMs can enhance the molecular inhibitory efficiency of iodobenzohydrazides against coxsackievirus B3.

Finally, the RT is finished with a mini-review by Gil and Carbó from the Universidad de Zaragoza and Universitat Rovia i Virgili (Spain), respectively. The authors highlight the relevance of the combination of molecular modulations simulations with quantum mechanics/molecular mechanics methods and theoretical calculations on cluster models. These calculations are starting to shed light on the factors governing the activity and selectivity for the hydrolysis of peptide and phosphoester bonds catalyzed by POMs. The authors have previously experience on theoretical calculations in cluster models (Solé-Daura et al., 2020). Moreover, the phosphoester bond hydrolysis catalysed by molybdate anions as artificial phosphoesterases has been also studied computationally (Lanuza et al., 2021; Martins et al., 2021; Sánchez-González et al., 2021). POMs as artificial enzymes have been tested by the group of Parac-Vogt, using POMs as catalysts in the hydrolysis of peptide bonds (Absillis and Parac-Vogt, 2012). Further computational studies have focus on the characterization of the reaction mechanism and the rationalization of the observed selectivity (Jayasinghe-Arachchige et al., 2019; Ly et al., 2019). In sum, the authors of the present mini-review are confident that in the coming years the computational studies on the biological activity of POMs will be an emergent research topic.

Altogether, the present RT reflects emergent 21st century applications of POMs, namely anticancer, antidiabetic and antiviral activities besides the applications of POMs as sensors and the contribution of computational studies for the understanding the biological activities of POMs. Within this RT a total of 25 authors were involved, where the majority are young researchers, The future is bright for POMs!
